# Abstract words processing induces parasympathetic activation: A thermal imaging study

**DOI:** 10.3389/fpsyg.2022.932118

**Published:** 2022-10-31

**Authors:** Melania Paoletti, Chiara Fini, Chiara Filippini, Giovanna M. Massari, Emilia D’Abundo, Arcangelo Merla, Francesca Bellagamba, Anna M. Borghi

**Affiliations:** ^1^Department of Dynamic and Clinical Psychology, and Health Studies, Sapienza University of Rome, Rome, Italy; ^2^Department of Neurosciences and Imaging, University G. d’Annunzio of Chieti-Pescara, Chieti, Italy; ^3^Department of Engineering and Geology, University G. d’Annunzio of Chieti-Pescara, Pescara, Italy; ^4^Institute of Cognitive Sciences and Technologies, National Research Council (CNR), Rome, Italy

**Keywords:** concreteness effect, language acquisition, development, thermography, reaction times, lexical decision

## Abstract

Abstract words (e.g., freedom) compose a significant part of speech. Despite this, learning them is complicated. Abstract concepts collect more heterogeneous exemplars and are more detached from sensory modalities than concrete concepts. Recent views propose that, because of their complexity, other people are pivotal for abstract concepts’ acquisition and use, e.g., to explain their meaning. We tested this hypothesis using a combined behavioral and thermal imaging paradigm. Twenty-one Italian children (10\F, mean age: 6 years) determined whether acoustic stimuli (concrete and abstract words; non-words) were or not correct Italian words (lexical decision). Concrete terms yielded faster responses than abstract ones: for the first time, this effect appears with response times in very young children. More crucially, the higher increase in temperature of the nasal tip (i.e., typically associated with parasympathetic dominance of the neurovegetative response) suggests that, with abstract concepts, children might be more socially and cognitively engaged.

## Introduction

Abstract concepts (e.g., “freedom”) are generally acquired later and through the linguistic rather than the perceptual modality (Villani et al., 2019), and are typically less iconic (Lupyan and Winter, 2018) as compared with concrete concepts (e.g., “table”). One of the most sound effects in the literature, the so-called concreteness effect, consists of an advantage in processing time and recall of concrete over abstract concepts (Paivio, 1990). The effect is very reliable in adults, but to our knowledge, no study so far has demonstrated it with response times in children. This effect testifies the complexity of abstract concepts.

Recent theories propose that compared to concrete concepts, which elicit more sensorimotor experiences, abstract ones evoke more linguistic, emotional, and social experiences (Borghi et al., 2017). Specifically, abstract concepts would be more grounded in language and sociality than concrete concepts (Borghi et al., 2019; Dove, 2019): because of the heterogeneity of their members, we would need more others to acquire them and understand their meaning (Borghi et al., 2019). According to a different perspective, using abstract concepts enhances social cohesion because defining beliefs in terms of intangible ideas might make social groups more cohesive (Gilead et al., 2020).

Still, the evidence on social-linguistic interaction’s relevance for abstract concept acquisition and representation is limited to a few studies with infants and adults. An experiment on early development showed that comprehension of infants’ first abstract concepts occurs at 10 and 14 months, parallel with the emergence of social cognition abilities like gaze following and joint action (Bergelson and Swingley, 2013). Feature production tasks with adults revealed that with abstract concepts, participants produced more features linked to social aspects of situations than with concrete concepts (Barsalou and Wiemer-Hastings, 2005).

Recent kinematics evidence with adults corroborates the idea that abstract concepts may strengthen collaboration. Participants showed more motor coordination when interacting with a confederate, who previously provided them with suggestions to guess abstract as compared with concrete concepts evoked by images in a conceptual guessing task (Fini et al., 2021).

Even if current evidence is scattered, demonstrating the strict linkage between abstract concepts and sociality would represent a crucial advancement for current theories on abstractness.

Our study investigates this issue in children using thermal imaging. Thermal infrared (IR) imaging allows a non-invasive recording of the cutaneous temperature and its topographic distribution by measuring the spontaneous body thermal irradiation. By recording the temperature dynamics in specific facial regions of interest (ROIs), it is possible to identify peculiar features correlated to emotional state and measures associated with standard physiological signals of the sympathetic and parasympathetic activity (Filippini et al., 2020).

Due to the strict correlation between the ANS activity and the skin temperature variation in some regions of interest (ROI), many studies have explored the use of thermal IR imaging to differentiate affective states and identify physiological correlates of emotional response (Kosonogov et al., 2012; Filippini et al., 2020).

For thermal inference of affective nature, the human face is significant since it can be easily recorded and is naturally exposed to social and emotional interaction. The nasal tip area resulted in being the most reliable region for detecting autonomic activity (Ioannou et al., 2014). Besides, evidence has shown that, on the occasion of sympathetic activation, the nasal tip area’s temperature decreased, attributing this effect to vasoconstrictive mechanisms and emotional sweating (Ioannou et al., 2014). On the other hand, studies revealed that the parasympathetic activation, which predominates in rest or pro-social activity, leads to vascular relaxation accompanied by a gradual temperature rise (Aureli et al., 2015). Therefore, thermal imaging is a suitable instrument to test whether abstract concepts’ processing elicits prosocial behavior that is also related with increased parasympathetic arousal and relaxation.

In our study, 5–7 year-olds were required to perform a lexical decision task. We measured their response times to the words and the facial temperature. We formulated two hypotheses, one pertaining to the response times, the other the thermal imaging measures:

1. If abstract words are more complex than concrete ones, children should find them harder to process and require more time to respond. Hence, we should find the concreteness effect in our sample.
2. If abstract words enhance prosocial behavior then the temperature of the nasal tip of children, which is associated with parasympathetic dominance, should increase more during abstract than during concrete concepts processing.

## Materials and methods

### Participants

Twenty-one Italian children (11 males and 10 females), aged between 5 and 7 years (mean age: 6 years and 29 days; SD: 0.69) with typical development, participated in the experiment.

The choice of the sample size is given by reference to previous similar studies in the literature (Ioannou et al., 2014; Aureli et al., 2015). As reported by the sociodemographic questionnaire filled by parents, all participants were residents of Rome, Italian mother tongue, and right-handed. Fifteen of them attended the last year of kindergarten, whereas the rest attended the first year of primary school.

No one reported any particular health problems or hospitalizations, except for two children: one suffered from migraine and the other did not specify the kind of health issue.

Participants were recruited by the experimenters distributing flyers at the local children’s library, at school entrance, and through social networks. A fundamental help in the recruitment was provided by the Montessori kindergarten teachers in San Lorenzo, in Rome, who encouraged the parents to let their children participate.

At the end of the experiment, children received a customized notebook and a certificate of participation as a gift.

Parents signed a written informed consent form, including a detailed description of the experimental procedure and the possible risks and side effects. The Ethics committee of the Department of Dynamic and Clinical Psychology, and Health Studies, of Sapienza, University of Rome, in accordance with the ethical standards of the 2013 Declaration of Helsinki, approved the experimental protocol.

### Material and task

Participants were tested individually, in a single experimental session lasting about 30 min, at the Infant Laboratory of the Department of Dynamic and Clinical and Health Psychology, set up in order to maintain the optimal environmental conditions for recording with an infrared camera. We ensured that during all the experimental sessions there were no direct sources of heat or ventilation, and the room temperature was kept constant, between 20 and 22 degrees, as well as the humidity level was around 40%, according to the International Academy of Thermology (IACT) guidelines (IACT, 2002).

Three experimenters were present in the laboratory room, each one with different commitments: the first one was in charge to record the experiment with the infrared camera, the second one to record the experiment with a digital camera, and the third one to administer the experimental task and the Italian version of the Peabody Picture Vocabulary Test (PPVT – Stella et al., 2000), which provided a measure of children’s receptive vocabulary ability. All participants exceeded the threshold of critical items identified by the test in function of their chronological age, except for one kid who did not complete the task.

A rectangular desk (100 × 80 cm) and two chairs, one for the child and the other one for the experimenter, were located on the side of the room.

At a distance of 1 m, we positioned the infrared camera aimed at the child’s face on a tripod stand and connected it to a pc on another desk where the thermographic measures were acquired. On the other side of the room, a children’s play table with little chairs was located. On it, one experimenter took care to put toys, pencils, and sheets of paper.

At the beginning of each experimental session, the experimenters invited the child and the parent to enter the laboratory room, made sure they were comfortable, and asked the mother to sign the consent form while another experimenter welcomed the child and offered him/her to make a drawing and/or to choose a toy. When the child seemed comfortable, the parent was asked to leave the room, and the same experimenter continued to play with the child for a few minutes. Then, she asked the child whether he/she wanted to play a game on the computer. The familiarization phase lasted on average around 15 min for each participant and allowed the child to be at ease. This time window was also crucial to stabilize the child’s basal temperature inside the laboratory room.

The child was comfortably seated in front of a rectangular table and watched a 1.366 × 768 resolution LCD monitor placed on the table at a distance of 60 cm from the eyes. A pillow was eventually placed on the chair where the child was sitting to let him/her be more comfortable and align the eyes height with the pc monitor. The experimenter was sitting next to the child and read to her/him the written instructions on the monitor. Before starting the experiment, the child was invited to avoid as much as possible any head movements and to avoid touching the face with the hands. The child was asked to perform a lexical decision task, administered on a PC controlled by E-Prime software (Version 3).

#### Experimental task

The child had to decide whether a series of pre-recorded words were Italian ones or not. If they knew the word, the children had to press a green button on the laptop keyboard; if they did not know the meaning of the word just listened, they had to refrain from pressing the button (go-nogo task).

A total of 40 words (16 concrete, 16 abstract, and 4 no words repeated twice) were presented by a synthetic pre-recorded audio. The task was structured in blocks (abstract/concrete); thus all the concrete words were introduced randomly and consecutively as well as the abstract ones. The order of block presentation was counterbalanced between participants.

Concrete and abstract words were selected from the databases of Della Rosa et al. (2010) and Villani et al. (2019), (see Table 1), and were matched for age of acquisition (AoA), familiarity, and word length (see Table 2). The abstract words included four categories of concepts identified as clusters in Villani et al. (2019): (i) philosophical-spiritual (e.g., “sogno”); (ii) physical, spatio-temporal, and quantitative concepts (e.g., “numero”); (iii) self-sociality concepts (e.g., “amicizia”), and (iv) emotive, inner states concepts (e.g., “bugia”).

**Figure 1:**
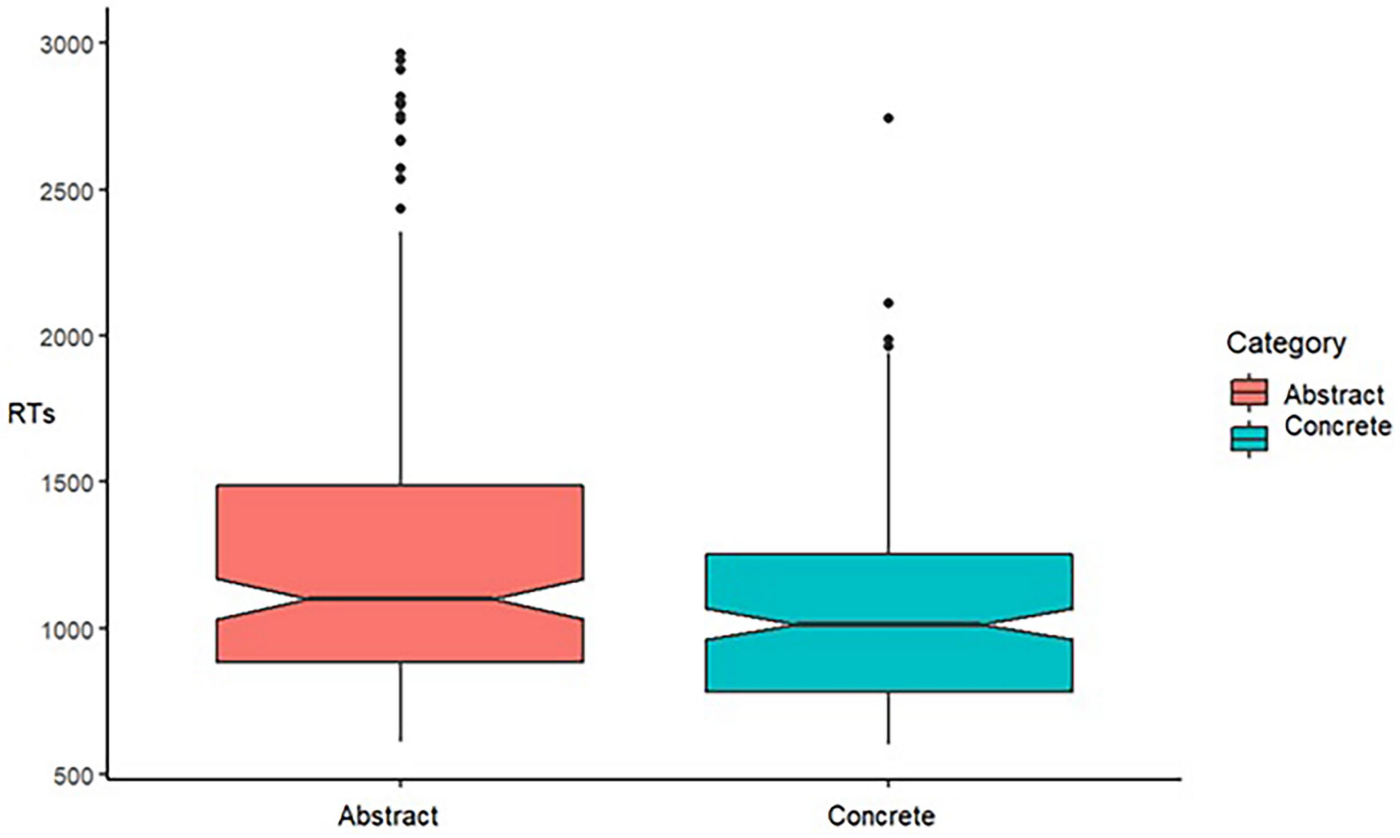
The analysis on RTs yielded a significant main effect of the Type of Concept (Abstract, Concrete), [*F*(1, 523) = 10.28, *p* = 0.0014]. Concrete concepts are processed faster than Abstract concepts. Horizontal lines in the boxes indicate the median, upper and lower borders indicate I and III quartile, “whiskers” extend to the farthest points that are not outliers, dots represent outlier trials.

**Table 1:** List of selected words and pseudowords.

Concepts			
Abstract		Concrete	
Attimo	Moment	Bandiera	Flag
Vergogna	Shame	Quercia	Oak
Amicizia	Friendship	Ombrello	Umbrella
Capriccio	Whim	Lampada	Lamp
Idea	Idea	Elicottero	Helicopter
Numero	Number	Sveglia	Alarm Clock
Ombra	Shadow	Tavolo	Table
Luce	Light	Poltrona	Armchair
Dispetto	Mischief	Pennello	Brush
Famiglia	Family	Statua	Statue
Festa	Party	Martello	Hammer
Bugia	Lie	Stivale	Boot
Gentilezza	Kindness	Palude	Swamp
Favola	Tale	Orologio	Clock
Fantasia	Fantasy	Muscolo	Muscle
Sogno	Dream	Trattore	Tractor
No Words			
Animode			
Bapana			
Bonago			
Melide			

**Table 2:** Dimensions of abstract and concrete selected words. concrete and abstract concepts did not differ for familiarity, age of acquisition, and word length dimensions.

**Dimensions**	**Abstract Concepts**	**Concrete Concepts**	**T-test**
Age of acquisition (AoA)	mean = 255 dev st = 34.25	mean = 241.42 dev st = 52.56	t(30) = 0.86, *p* = 0.393
Word length	mean = 6.56 dev st = 1.86	mean = 7.62 dev st = 2.03	t(30) = −2, *p* = 0.055
Abstractness	mean = 374.06 dev st = 112.25	mean = 114.64 dev st = 20.06	t(30) = 8.17, p < 0.001
Concreteness	mean = 438.75 dev st = 80.11	mean = 689.83 dev st = 20.76	t(30) = −11.7, *p* < 0.001
Modality of acquisition (moa)	mean = 282.5 dev st = 61.23	mean = 205,34 dev st = 64.31	t(30) = 3.47, p < 0.001
Imageability	mean = 572.18 dev st = 53.81	mean = 673.97 dev st = 26.29	t(30) = −6.80, p < 0.001
Contextual availability	mean = 595.53 dev st = 67.12*	mean = 636.90 dev st = 36.63	t(30) = −2.22, *p* < 0.034

The child was instructed to respond as quickly and accurately as possible. Each trial started with the presentation of a fixation cross lasting 8,000 ms followed by a voice audio-clip pronouncing the word, lasting 1,000 ms. Between the two blocks, 1/2 min time was allotted for rest. Before the task began, a training phase was administered in which the child was presented with one abstract and one concrete word.

### Thermal data acquisition and processing

Infrared thermography, using the thermal radiation naturally emitted by the body, enables the measurement of the skin temperature in a non-invasive and eco-sustainable way. Hence, it is especially suited to integrate physiological elements in research on socio-emotional development, especially in children.

In order to investigate the autonomous response of children to the stimuli presented in the experimental task, the child’s facial temperature was recorded through an infrared thermal camera, the FLIR A655sc model. In particular, it was characterized by a 25° lens, a matrix of 640×480 bolometric sensors, a full frame sampling of 50 Hz, and a calibration range between −40° and 650°. The camera was blackbody-calibrated to remove noise-effects related to the sensor drift/shift dynamics and optical artifacts. The sampling rate for thermal imaging was set at 10 frames/s. The thermal camera was associated with a Logitech C920 webcam, located above it for the acquisition of the digital videos, and with the IRI Image PRO 2.0 software, developed by Next2U s.r.l (Pescara Italy)^1^ and described in Cardone et al. (2021), for the coding of the thermal signals. The thermal video processing performed through the IRI Image PRO 2.0 software relied on the following procedures: (1) Facial landmarks’ automatic recognition in the visible domain using the OpenFace library. OpenFace is an open-source tool, able to detect facial landmarks, and recognizes facial action units. (2) Identification of the regions of interest on the participant’s face with respect to the facial landmarks. (3) Co-registration of the selected region of interest in the visible video with the corresponding region in the thermal video. The co-registration was performed by applying a geometric transformation of the visible coordinates (calculated based on different fields of view), resolution, and position. Furthermore, during the tracking phase, it was necessary to optimally calibrate the positions of the various regions of interest on the participant’s face, in order to correctly trace the thermal signal throughout the test. Finally, an editing process permitted verifying the quality of the tracing frame by frame, allowing the experimenter to correct it if this was not satisfying, due to the frequent movements of children during the test. Five children were excluded from the statistical analysis due to technical reasons: (i) the incorrect positioning of the thermal camera during the recording of the task which did not allow to exactly detecting their facial temperature (N = 1); (ii) the malfunction of the thermal camera before starting the experiment (N = 2); and (iii) the malfunction of the software when extracting the facial temperature from thermal videos (N = 2).

## Results

### RTs data analysis

One participant among the 21 recruited was removed from the analysis because he did not perform the task as required from the instructions. A total of 640 trials—320 concrete and 320 abstract (two blocks composed of a total of 32 words, 16 abstract, 16 concrete words, and 4 pseudowords repeated twice, collected in 20 participants) were included in the analysis—after discarding 160 trials of pseudowords. The percentage of the given responses was higher in the abstract words compared (294 over 320 trials, 92%) with the concrete ones (272 over 320 trials, 85%). We included in the analysis of RTs only participants with a percentage of given responses above 2 SD from the average percentage of responses. After having discarded two participants below 2 SD from the average percentage of given responses, the trials were 525. The residuals were not normally distributed (W = 0.76, *p* = 0.001), as we have testified by applying the function “qqmath” requiring the “Lattice” package (Sarkar, 2008) on the Linear mixed-effects models which included as dependent variable RTs, as a fixed factor the Category (abstract, concrete), and as random intercepts participants and words. Then, we opted for applying the Generalized Least squares (gls) model with the “nlme” package (Pinheiro et al., 2012). The Generalized Least Squares model included as dependent variable RTs and as fixed factor the Category (abstract, concrete). A significant effect of the Category was obtained [*F*(1,523) = 10.28, *p* = 0.0014], showing that concrete words (RTs = 939, SE = 48.6) were processed faster than abstract ones [(RTs = 1.162, SE = 50.2) see Figure 1]. Overall, participants were faster with the concrete words compared to the abstract ones. Such a result extends the concreteness effect, largely documented in adults, to children.

**Figure 2:**
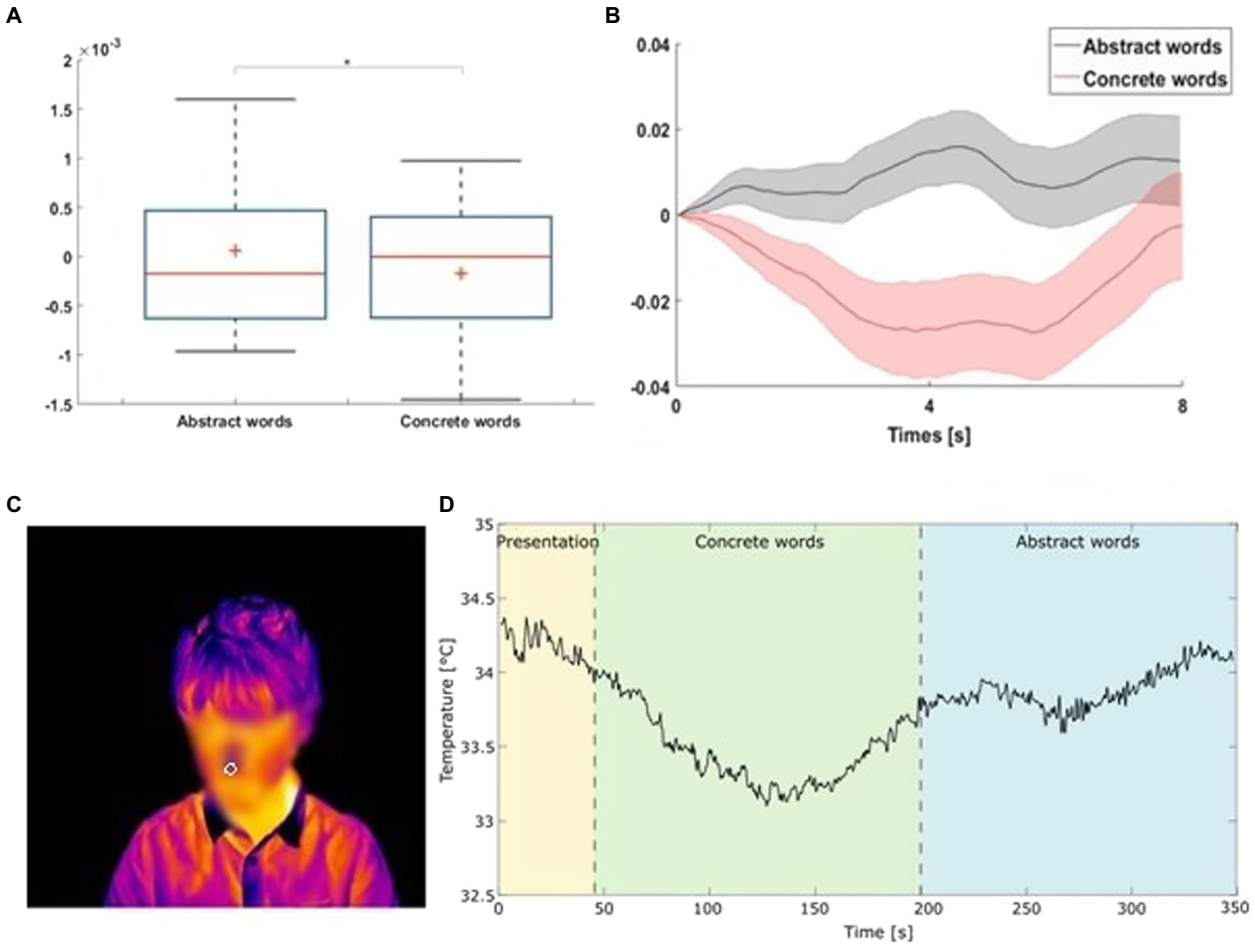
Panel **(A)** Slopes of the nasal tip temperature during abstract and concrete word presentation. Statistical analysis resulted in a significant difference between the two conditions [*F*(1,461) = 4.108, *p* = 0.0432]. Listening abstract words induced an increased (positive) slope of the thermal signal in comparison to concrete words. Horizontal lines in the boxes indicate the median, upper and lower borders indicate I and III quartile, “whiskers” extend to the farthest points that are not outliers, dots represent outlier trials. Panel **(B)** Average nasal tip temperature trend during abstracts and concrete word conditions across repetitions. The shadowed colored areas represent the standard error of each curve. Panel **(C,D)** Thermogram of a representative participant: ROI placement on the nose tip (white circle) and the associated thermal signal after being visually inspected and corrected for motion artifacts. The first dashed line represents the beginning of the trial relating to concrete words and the second one represents the end of the concrete words and the beginning of abstract words.

### Thermal data analysis

The statistical analysis focused on temperature variations that occurred on the nasal tip, which has proved to be one of the most reliable regions for detecting physiological activation related to the autonomic nervous system activity (Ioannou et al., 2014; Aureli et al., 2015).

The thermal signal was further corrected from residual motion artifacts. We identified and replaced motion errors using a linear interpolation of the value neighboring the artifact. Then we applied a third-order Butterworth low-pass filter (1 Hz) in order to reduce high-frequency noise. Prior to feature extraction, the thermal signal was z-score normalized.

Then, we computed the slope of the thermal signal of each stimulus, which represented the dependent variable. Indeed, the slope, which is described by the signal’s first-time derivative, provides information about how quickly the temperature change over time and has been used in literature to infer autonomic nervous system activity in 4 to 5 years old children (Filippini et al., 2021). On 463 trials, we run a Generalized Least squares (gls) model, since the residuals were not normally distributed (W = 0.90, *p* = 0.001), as we have testified by applying the function “qqmath” requiring the “Lattice” package (Sarkar, 2008) on the Linear mixed-effects model which included as dependent variable the slope of the thermal signal, as fixed factor the Category (abstract, concrete), and as random intercepts participants and words.

As predicted, we obtained a significant effect of the Category [*F*(1,461) = 4.108, *p* = 0.0432]. Tukey *post-hoc* comparisons indicated that listening abstract words (0.0000724, SE = 0.0000738) induced an increased (positive) slope of the thermal signal in comparison to concrete words [−0.000137, SE = 0.0000752, t(440) = 2.024, *p* = 0.0435; Figure 2].

## Discussion

The results revealed that children process concrete faster than abstract concepts in a lexical decision task. The finding replicates the well-known concreteness effect (Paivio, 1990), showing it for the first time in preschool-aged children with response times. A recent study shows a concreteness effect in children aged 6 to 11 years, but the results pertain to accuracy, not response times (Ponari et al., 2018).

Thermal IR imaging results revealed that children’s nose tip temperature increased during abstract compared to concrete word processing. The detected thermal variation suggests a change in the children’s autonomic system, indicating a dominance of the parasympathetic component. Unlike the sympathetic component, which predominates in a stressful situation, the dominance of the parasympathetic branch of the nervous system implies higher social and cognitive engagement and relaxation. Indeed, this component mediates the children’s positive engagement with persons and objects (Porges, 2007; Aureli et al., 2015). Thereby, the increase in temperature with abstract concepts is compatible with the hypothesis we advanced. A major social and cognitive engagement with abstract concepts might be related with the idea that children need to rely more on other people with abstract concepts than concrete ones. As we recently proposed, the processing of abstract concepts may be strictly linked to metacognition. When we process abstract concepts, we might be more uncertain and less confident to know the exact word meaning. This higher uncertainty may be one of the causes of longer response times with abstract concepts and explain the concreteness effect. Consistently, the longer processing time of abstract concepts might be due to the necessity to keep them in the phonological working memory; this would lead to the engagement of the left inferior frontal gyrus, the neural area more engaged during abstract concepts processing (Desai et al., 2018). Hence, abstract concepts would be monitored longer than concrete ones, due to their higher complexity. The monitoring process might have two possible outcomes that might occur sequentially or not. First, it might lead to a more extended inner search for meaning. Second, it might lead to relying on others to get support (social deference; Shea, 2018; Borghi et al., 2019, 2021). Overall, the results are compatible with the proposal that abstract words, because of their complexity and heterogeneity of their members, might promote a major social and cognitive engagement during their acquisition and processing. This engagement might be expressed by parasympathetic dominance. We cannot exclude that our results might be partially driven by a stronger emotionality intrinsic to abstract words, such as of arousal and valence, compared to concrete words. Future studies with larger sample sizes and multiple methodological approaches are needed to deepen the link between sociality, affect, and abstract concepts processing.

## Data availability statement

The datasets presented in this study can be found in online repositories. The names of the repository/repositories and accession number(s) can be found at: https://osf.io/n8bu4/.

## Ethics statement

The studies involving human participants were reviewed and approved by the Ethics Committee of the Department of Dynamic and Clinical Psychology, and Health Studies, of Sapienza, University of Rome, in accordance with the ethical standards of the 2013 Declaration of Helsinki. Written informed consent to participate in this study was provided by the participants’ legal guardian/next of kin.

## Author contributions

AB, FB, AM, CFin, and MP: conceptualization. MP, CFin, GM, ED, and CFil: data curation. CFin and CFil: formal analysis. MP, CFin, GM, and ED: investigation. AB, FB, AM, CFin, and MP: methodology. AB, FB, and AM: supervision. AB, FB, AM, CFin, MP, and CFil: writing. All authors contributed to the article and approved the submitted version.

## Funding

This research was funded by the European Union’s Horizon 2020 research and innovation programme – TRAINCREASE “From Social Interaction to Abstract Concepts and Words: Towards Human-centered Technology Development” (Proposal no. 952324) and by Sapienza Excellence projects – “Concepts in interaction with others and with ourselves: abstractness in social interaction, metacognition and mind wandering” (grant no. RG12117A5D1EB0B3).

## Conflict of interest

The authors declare that the research was conducted in the absence of any commercial or financial relationships that could be construed as a potential conflict of interest.

## Publisher’s note

All claims expressed in this article are solely those of the authors and do not necessarily represent those of their affiliated organizations, or those of the publisher, the editors and the reviewers. Any product that may be evaluated in this article, or claim that may be made by its manufacturer, is not guaranteed or endorsed by the publisher.

## Footnotes

^1^ www.next2u-solutions.com
